# High Intensity Long Interval Sets Provides Similar Enjoyment as Continuous Moderate Intensity Exercise. The Tromsø Exercise Enjoyment Study

**DOI:** 10.3389/fpsyg.2019.01788

**Published:** 2019-08-06

**Authors:** Edvard H. Sagelv, Tord Hammer, Tommy Hamsund, Kamilla Rognmo, Svein Arne Pettersen, Sigurd Pedersen

**Affiliations:** ^1^School of Sport Sciences, Faculty of Health Sciences, University of Tromsø the Arctic-University of Norway, Tromsø, Norway; ^2^Department of Psychology, Faculty of Health Sciences, University of Tromsø the Arctic-University of Norway, Tromsø, Norway

**Keywords:** training, aerobic, affect, public health, emotion

## Abstract

**Objective:**

High intensity interval exercise sessions with interval sets over 3 min may provide superior cardiorespiratory fitness benefits. To our knowledge, the exercise enjoyment of interval sets over 3 min is not yet elucidated. The aim of this study was to examine exercise enjoyment following one session with four intervals of 4 min high intensity exercise (HIIE) versus one session of 45 min moderate intensity continuous exercise (CE) in iso-caloric conditions using a randomized crossover design.

**Methods:**

Seven young healthy participants were recruited to undergo two different exercise sessions in a randomized order: (1) 4 × 4 min intervals at >90% of maximum heart rate (HR_*max*_) with 3 min of rest between interval sets, and (2) 45 min CE at 70% of HR_*max*_. Peak oxygen uptake and HR_*max*_ were evaluated prior to the experiment. The participants reported their perceived exercise enjoyment using the 18-item physical activity enjoyment scale (PACES) questionnaire and their rating of perceived exertion (RPE) using Borg’s 6–20 scale.

**Results:**

There was no difference in the PACES score between the high intensity interval exercise session [median: 95.5 (inter-quartile range: 21.8)] and the moderate intensity CE session [91.0 (13.5), *p* = 0.36, *r* = −0.22]. The participants reported a higher RPE in the high intensity interval exercise session [16.5 (2.0)] compared with the CE session [9.0 (2.0), *p* = 0.01, *r* = −0.88].

**Conclusion:**

Similar exercise enjoyment was reported following four high intensity intervals of 4 min compared with a moderate intensity CE session in this randomized crossover study with iso-caloric conditions. If enjoyment is a mediating factor for engaging in exercise, one should expect a similar probability of exercise adherence following high intensity 4 min intervals and continuous moderate intensity exercise when prescribing aerobic exercise as preventive medicine.

## Introduction

Although aerobic exercise is an effective means for improving cardiorespiratory fitness (Bacon et al., 2013), preventing premature mortality (Blair et al., 1995; Myers et al., 2002; Lear et al., 2017), and preventing and rehabilitating from various health conditions and diseases (Blair et al., 1995; Lee et al., 1995; Kruk, 2007; Pedersen and Saltin, 2015), adherence to exercise programs is low and seems to be dependent on multiple personal and demographic factors (Leslie et al., 1999; Stutts, 2002; Trost et al., 2002; Sequeira et al., 2011; Picorelli et al., 2014). For the individual, the main reported factors inhibiting adherence are lack of time and enjoyment (Leslie et al., 1999; Stutts, 2002), which have led researchers to design exercise sessions with improved dose-response effects and increased time efficiency in an attempt to provide individuals with alternatives to overcome these barriers (Oliveira et al., 2018).

High intensity interval exercise sessions have been suggested to be effective both in terms of dose-response effects and time efficiency (Oliveira et al., 2018). Additionally, high intensity interval exercise sessions seem to provide superior cardiorespiratory fitness improvements compared with continuous exercise (CE) (Bacon et al., 2013); thus, exercise of this format and intensity seems applicable for both health improvements and exercise adherence. Exercise adherence may partly depend upon the enjoyment experienced during or after exercising (Raedeke, 2007), as behavior providing increased enjoyment may result in a higher probability of adherence, and lower enjoyment may result in a lower probability of repeating the behavior (Kahneman et al., 1993; Zenko et al., 2016). Although not always consistent (Oliveira et al., 2013; Martinez et al., 2015; Decker and Ekkekakis, 2017), the exercise enjoyment measured with the physical activity enjoyment scale (PACES) questionnaire (Kendzierski and DeCarlo, 1991) is usually reported to be higher following high intensity interval exercise compared with moderate intensity CE (Oliveira et al., 2018). Interestingly, the observation of higher enjoyment is also present when rating of perceived exertion (RPE) is higher in the high intensity intervals compared with moderate intensity CE (Oliveira et al., 2018). Based on RPE, it seems that individuals prefer exercises that are intermittent in nature compared with CE, which is monotounuous in nature (Coquart et al., 2008a,b), and this may also explain why higher enjoyment is reported for high intensity intervals. However, multiple high intensity interval exercise session designs are found in the studies investigating exercise enjoyment (Oliveira et al., 2018), which limits their comparability. These differences include variations in interval duration, ranging from seconds (Price and Moss, 2007; Wiewelhove et al., 2016; Warr-di Piero et al., 2018), to 1 min (Jung et al., 2014; Heisz et al., 2016) and bouts of up to 3 min (Bartlett et al., 2011; Decker and Ekkekakis, 2017); a previous study reported that longer interval sets are less enjoyable compared with those of shorter durations (Martinez et al., 2015).

It has previously been suggested that exceeding the anaerobic threshold, defined as the ventilatory anaerobic threshold, is the main physiological marker for negative affective responses to exercise (Bartlett et al., 2011; Oliveira et al., 2018). Over the past few decades, many anaerobic threshold definitions have been suggested, all of which correspond to different exercise intensities (Hall et al., 2002; Faude et al., 2009). Another definition of the anaerobic threshold, the lactate threshold (Faude et al., 2009), is suggested to reflect and even cause the ventilatory anaerobic threshold (Wasserman et al., 1973; Davis et al., 1976; Yoshida et al., 1987; Ghosh, 2004; Faude et al., 2009). In fact, higher blood lactate concentrations are observed following intervals of longer duration compared with those of shorter duration (Beaver et al., 1986; Price and Moss, 2007; Kilpatrick et al., 2015a; Wiewelhove et al., 2016; Warr-di Piero et al., 2018); consequently, the anaerobic contribution seems to be a mediating factor for exercise enjoyment, both when defining the anaerobic threshold using the ventilatory and lactate measures. Thus, these findings suggest that interval durations should be short (<1 min) to elicit exercise adherence (Rhodes et al., 2009; Jekauc, 2015; Kilpatrick et al., 2015a,b).

At the same time, the greatest improvements in cardiorespiratory fitness following high intensity interval exercise are observed with interval set durations over 3 min, which is explained by central adaptations in the heart (Bacon et al., 2013). In contrast, short durations seem to mainly elicit peripheral adaptations in the muscles (Sloth et al., 2013). Considering that stroke volume of the heart is the limiting factor for cardiorespiratory fitness (Bassett and Howley, 2000), interval sets over 3 min may have clinically relevant implications for public health trajectories.

Recently, one study reported lower blood lactate concentrations and less post-exercise oxygen uptake following 4 min intervals (>90% of peak heart rate) compared with supramaximal 20 s sprints (maximal effort) (Valstad et al., 2018), indicating a higher anaerobic contribution with supramaximal sprints compared to 4 min intervals, which is supported by studies comparing the anaerobic contribution of supramaximal sprints with interval durations up to 2 min (Wiewelhove et al., 2016; Wood et al., 2016). Hence, recent findings of higher blood lactate concentrations following short interval exercise compared with intervals over 3 min (Bassett and Howley, 2000), along with data implicating the anaerobic threshold as the mediator for negative affective responses to exercise (Hall et al., 2002; Oliveira et al., 2018), suggest that the reduced enjoyment associated with increased interval duration (Martinez et al., 2015) may have been misinterpreted in the field. Considering that high intensity interval exercise sessions with interval sets over 3 min may provide superior cardiorespiratory fitness benefits, it is necessary to investigate factors related to exercise adherence in such sessions with long interval durations.

Thus, the aim of this study was to assess perceived exercise enjoyment following one session of high intensity intervals of 4 min each compared with one session of moderate intensity CE using an iso-caloric, randomized crossover design.

## Materials and Methods

### Participants

Eight young healthy participants (4 females and 4 males) were recruited, of which one (male) withdrew prior to completion of the study (reported reason: lack of time), resulting in seven participants (4 females and 3 males) for the final analyses (Table 1). All participants defined themselves as recreationally active by answering “active” on the following question: “*Do you consider yourself to be physically active or inactive?*” The participants were recruited by posters on the university campus. Prior to participation and before providing verbal and written informed consent, the participants were informed verbally and in writing about the purpose of the study and were informed of their right to withdraw from the study at any time without providing any reason. The study was carried out in accordance with ethical standards for health research under the Declaration of Helsinki, and the Norwegian Social Science Data Services approved the study in addition to the storage of personal data (Approval reference number: 57360). Further approval from a Regional Ethics Committee was not required for this study as per applicable institutional and national guidelines and regulations for sport and exercise science.

**TABLE 1:** Participant characteristics (*n* = 7).

Age (year)	24 (3)
Weight (kg)	70.7 (7.5)
Height (cm)	173 (12.5)
Body mass index (kg m^–2^)	23.6 (1.1)
Peak oxygen uptake (ml kg^–1^ min^–1^)	53.2 (9.4)
Rating of perceived exertion at volitional fatigue	19 (1)

*Data are reported as the median (inter-quartile range).*

### Pre-exercise Tests

Prior to the two exercise sessions, the participants underwent a test to volitional fatigue while walking and running on a treadmill to determine cardiorespiratory fitness defined as peak oxygen uptake (VO_2*peak*_). Prior to the test, weight and height were measured using a portable scale (Seca 876, Seca GmbH & Co., KG, Germany) and a stable stadiometer (Seca 217, Seca GmbH & Co., KG, Germany), respectively, and body mass index (BMI) was calculated (kg m^–2^).

First, the participants warmed up on a treadmill (Woodway Ergo ELG 70, Waukesha, United States). For the first 5 min, the speed was determined by the participant after they were instructed to walk or run at a value of 10–14 on Borg’s RPE scale, indicating very light to somewhat hard effort (Borg, 1982), in order to make theparticipants familiar with the use of the RPE scale at moderate intensity and thus perceptual “exercise anchoring” the RPE measurements (Coquart et al., 2012). Thereafter, the speed was increased by 1 km h^–1^ for 2.5 min followed by another 1 km h^–1^ increase for 2.5 min, which was supervised by the instructor to ensure that the participants were properly warmed up prior to the test to volitional fatigue.

Prior to starting the test to volitional fatigue, the participants were equipped with a face mask (Cosmed Srl, Rome, Italy) connected to a portable cardiorespiratory analyser (K5, Cosmed Srl, Rome, Italy) set in mixing chamber mode and placed on the participant’s back. A heart rate (HR) monitor (Garmin HRM3, Garmin Ltd., Lathe, Kansas, United States) was strapped around the thorax and transferred the HR values to the cardiorespiratory analyser. When set in mixing chamber mode, the portable K5 analyser has been shown to provide valid results for tests to volitional fatigue compared to a previously validated stationary analyser (Perez-Suarez et al., 2018). Respiratory and HR values were recorded every 10 s. Prior to the test, the cardiorespiratory analyser was calibrated for oxygen and carbon dioxide using known gas concentrations of 16 and 5%, respectively. The inspiratory flow was manually calibrated against the turbine using a 3 l volume syringe (Calibration Syringe, Cosmed Srl., Rome, Italy). For monitoring the measured values during the entire test, the cardiorespiratory analyser was connected to a portable laptop (ThinkPad, Lenovo Group Ltd., Beijing, China) by Bluetooth and assessed using the software provided by the manufacturer (Cardiopulmonary diagnostics software, Cosmed Srl., Rome, Italy). At the start of the test, the treadmill was set to a fixed 5.3% incline and a starting speed of 5 km h^–1^, and the speed was increased every minute until reaching volitional fatigue. Every 30 s, the participants were asked if they could cope with a 1 km h^–1^ increase in 30 s, and they answered using a thumb up or down for yes and no, respectively. If they answered no, they were instructed to keep the pace until reaching volitional fatigue and then jump off the treadmill. VO_2*peak*_ was defined as the mean of the three highest consecutive 10 s oxygen uptake recordings (Howley et al., 1995), and HR_*max*_ was defined as the highest HR recording during the last minute of the test. Immediately following volitional fatigue, the participants rated their RPE, where fatigue was defined as RPE ≥ 17, which indicates a very hard effort (Borg, 1982).

### Exercise Sessions

Following the pre-exercise tests, the participants performed the following two exercise sessions in a randomized order while walking or running on the treadmill: (1) high intensity interval exercise: 4 repetitions of 4 min high intensity intervals at >90% of HR_*max*_, interspaced with a 3 min active recovery at 70% of HR_*max*_. This session was initiated with a 10 min warmup and ended with a 3 min cool down, both at 70% of HR_*max*._ (2) moderate intensity CE, with 45 min CE at 70% of HR_*max*_. Randomization of the first exercise session was performed using the Research Randomizer software (Urbaniak and Plous, 2013). Exercise intensity was monitored with an HR monitor (Polar M400, Polar, Oy, Finland) connected to a HR belt (H7, Polar, Oy, Finland) strapped around the participant’s thorax. The exercise sessions were matched for energy expenditure, which ensured iso-caloric conditions. Both exercise sessions were derived from Helgerud et al. (2007). A 7-day washout period was applied between the pre-test and both exercise sessions in order to avoid any carryover effect on perception of fatigue from the previous session.

### Measurement of Exercise Enjoyment and Perceived Exertion

The PACES questionnaire was used to assess exercise enjoyment (Kendzierski and DeCarlo, 1991). PACES is an 18-item scale consisting of statements on exercise enjoyment in which participants rate their agreement with each statement on a scale from 1 to 7 (Kendzierski and DeCarlo, 1991). The PACES questionnaire is reported to be valid for internal consistency (Cronbach’s alpha: 0.96) and reliable for repeatability (intraclass correlation coefficient: 0.93) in young adult women and men (Kendzierski and DeCarlo, 1991). The PACES questionnaire is originally in English; therefore, the PACES questionnaire was translated to Norwegian by two of the authors (ES and SP) prior to the study. Thereafter, the translated versions were compared, and divergent statement translations were discussed, along with cognitive debriefing of alternative translations, until final agreement on the wording was achieved. If no final agreement was reached, a third independent individual was asked to decide on the final wording. Following the translation into Norwegian, a native English-speaking individual with fluent Norwegian vocabulary back-translated the Norwegian version to English. The new English version was then compared to the original questionnaire. If a statement differed from its corresponding original statement following the back-translation, the discrepancies were carefully discussed. Thereafter, suitable adjustments were made after agreement. Finally, proofreading was performed to correct any minor errors (Wild et al., 2005). The translated version of the PACES questionnaire can be found in the Supplementary Table 1.

By replacing the phrase “*Please rate how you feel at the moment about the physical activity you have been doing*” with “…*the physical activity you are doing*,” the participants were asked to answer the questionnaire following 50% completion of each exercise session while still exercising (no break was given). For the high intensity interval session, the PACES was asked following interval set 2, which corresponds to 55% completion of the session, to avoid answering during a high intensity interval. The questionnaire was also filled out at the end of the exercise sessions using the original statement. An instructor read each statement item, and the participants rated it from 1 to 7 depending on their perception of the statement, where higher and lower scores could indicate higher exercise enjoyment depending on how the statement was phrased. Thereafter, a full score is calculated, with reversed negative items converted to positive values (i.e., if a low score indicated higher exercise enjoyment, it was converted to a positive value, with a score of 1 being converted to 7 in the calculation, for example). The lowest and highest possible score is 18 and 126 arbitrary units, respectively. Additionally, the participants were asked to rate their RPE from 6 to 20 on Borg’s scale (Borg, 1982) during the same exercise period as the PACES reporting. “Exercise anchoring” of the RPE measurements was used, where the participants was made familiar with the effort indicating low to moderate intensity and maximal intensity from the warmup stage and following volitional fatigue, respectively, in the test to volitional fatigue (Coquart et al., 2012).

### Statistical Analysis

The Statistical Package for Social Sciences (SPSS, Version 25, International Business Machines Corporation, United States) was used to perform all statistical analyses. The participant who withdrew from the study prior to completion was excluded from all analyses. Due to the small sample size, assumptions of normal distribution were considered inappropriate. Thus, the Wilcoxon signed-rank test was applied to test for differences in perceptual responses between the two exercise sessions, and all variables are presented as the median (inter-quartile range). A Supplementary Tables 2, 3 with means and standard deviations is available in the supporting information for potential future meta-analyses (Lydersen, 2015). A 7-day washout period was considered sufficient to avoid fatigue; thus, no statistical test for carryover effects between exercise conditions was applied. The intra-exercise and post-exercise data were compared together after pooling the 14 observations (seven observations each before and after exercise) and separately using each set of seven observations. Effect sizes were calculated as product-movement *r* with the following formula: *r* = z/**√**n, with *z* being the *z*-value of the Wilcoxon signed-rank test and ***√****n* being the square root of the number of observations. A product-movement r of 0.10–0.29, 0.30–49 and ≥0.50 indicated small, medium and large effect sizes, respectively (Cohen, 1992). The alpha level was two-tailed and set to ≤0.05.

## Results

**FIGURE 1:**
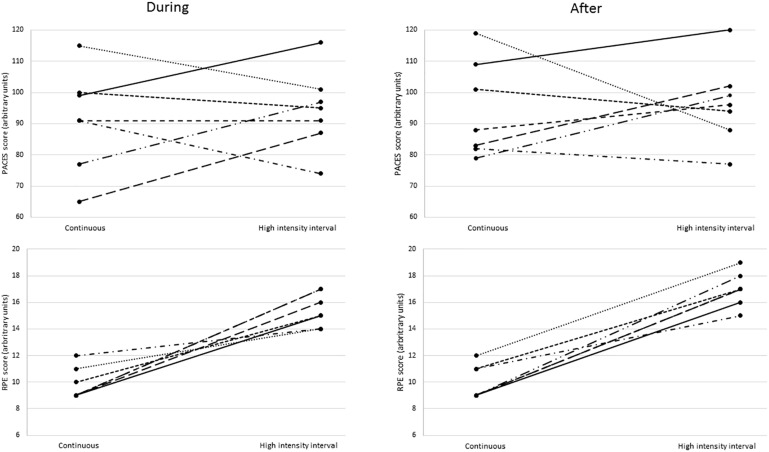
PACES scores. Individual perceptual scores in the moderate intensity continuous exercise session and the high intensity interval exercise session separately as “during” and “after” (*n* = 7). Each line represent one participant. PACES, physical activity enjoyment scale; RPE, rating of perceived exertion.

The participants’ perceptual responses are presented in Table 2, with the individual responses illustrated in Figure 1. The participants reported similar enjoyment following the high intensity interval exercise session and the moderate intensity CE session in both the pooled analysis, where the “during” and “after” exercise scores are pooled together (*p* = 0.36, *r* = −0.24), and in the separate analyses for “during” (*p* = 0.40, *r* = −0.32) and “after” the exercise sessions (*p* = 0.50, *r* = −0.26). There were no differences in PACES scores between “during” and “after” in either the high intensity interval exercise session (*p* = 0.24, *r* = −0.45) or the moderate intensity continuous session (*p* = 0.30, *r* = −0.38).

**TABLE 2:** Exercise Enjoyment and Rating of Perceived Exertion scores pooled (*n* = 14) and separate as “during” and “after” (*n* = 7) for high intensity interval exercise and moderate intensity continuous exercise.

	**High intensity interval exercise**	**Continuous exercise**
**Physical activity enjoyment scale**		
Pooled (*n* = 14)	95.5 (21.8)	91.5 (13.5)
During exercise (*n* = 7)	95 (14)	91 (23)
After exercise (*n* = 7)	96 (14)	88 (27)
**Rating of perceived exertion**		
Pooled (*n* = 14)	16.5 (2)^*^	9 (2)
During exercise (*n* = 7)	15 (3)^*^^#^	9 (2)
After exercise (*n* = 7)	17 (2)^*^	9 (2)

Data are shown as the median (inter-quartile range). ^*^Significant difference between high intensity interval exercise and continuous exercise <0.05. ^#^Significant difference between “during” and “after” exercise in the same exercise mode <0.05.

The participants reported higher RPE following the high intensity interval exercise session compared with the moderate intensity CE session in the pooled analysis (*p* = 0.01, *r* = −0.88), and in the separate analyses for “during” and “after” (both *p* = 0.02, both *r* = −0.90). The RPE was lower “during” the high intensity interval exercise session compared with “after” the high intensity interval exercise session (*p* = 0.02, *r* = −0.86) but did not differ between “during” and “after” in moderate intensity CE session (*p* = 0.56, *r* = −0.22).

## Discussion

In this randomized crossover design with iso-caloric exercise sessions, a session of four intervals of 4 min high intensity exercise interspaced with 3 min rest between interval sets was found to provoke higher perceived exertion but provided similar enjoyment compared with a session of moderate intensity CE.

The finding of similar enjoyment following a high intensity interval exercise session and a CE session is consistent with some studies (Hall et al., 2002; Thum et al., 2017; Vella et al., 2017), whereas others suggest higher enjoyment following high intensity intervals (Oliveira et al., 2013, 2018). Cardiorespiratory fitness and fat mass have been suggested as mediating factors for the inconsistent results; however, a recent meta-analysis reported non-linear associations between these factors and exercise enjoyment, suggesting the presence of other mediating factors for exercise enjoyment following different exercise modalities (Oliveira et al., 2018).

Furthermore, the present study’s results challenge the interpretation that increased duration of interval sets leads to lower perceived exercise enjoyment. Although we did not measure blood lactate concentration in the present study, a previous study reporting lower blood lactate concentration following interval sets greater than 4 min in duration (Valstad et al., 2018) compared with a substantially higher blood lactate concentration during supramaximal sprints (Wiewelhove et al., 2016; Warr-di Piero et al., 2018) may indicate a lower anaerobic contribution in interval set durations over 4 min compared with shorter interval set durations. Moreover, it has previously been suggested that negative affective responses are observed at supramaximal intensities (Ekkekakis et al., 2011). Thus, the relationship between perceived enjoyment and interval duration may be N-shaped; low at short sprint durations followed by higher exercise enjoyment at short duration (∼1 min) intervals with decreasing enjoyment as the duration increases up to 3 min, followed by increasing enjoyment when performing intervals over 3 min. Considering the potentially clinically relevant increase in stroke volume following intervals over 3 min (Bacon et al., 2013), this may present important considerations for exercise prescriptions of high intensity intervals. Nevertheless, in order to facilitate exercise adherence and because high intensity intervals and moderate intensity CE both improve cardiorespiratory fitness (Bacon et al., 2013), the preferred exercise modality of the individual should be the decisive factor when prescribing aerobic exercise as preventive medicine, as suggested previously (Ekkekakis et al., 2011).

RPE was higher both during and after the high intensity interval exercise session compared with the CE session. Exertion is anticipated to increase with increased effort (Borg, 1982). However, exercise intensity relative to the duration also effects exertion, which is more prominent when exceeding the anaerobic threshold (Garcin and Billat, 2001). Thus, although the exercise sessions was iso-caloric and the high intensity interval session was shorter, the accumulated exertion from both intensity and duration in the high intensity interval session is higher compared with the CE session at moderate intensity. To our knowledge, these data are in accordance with all previous research regarding effort and exertion in healthy individuals (Venhorst et al., 2018).

The participants were asked to answer the PACES scale questionnaire both during and after the exercise sessions in the present study. Originally, the PACES questionnaire was developed for use following exercise sessions (Kendzierski and DeCarlo, 1991). Thus, its use during the exercise sessions may have been inappropriate. Ideally, the Exercise Enjoyment questionnaire (Hardy and Rejeski, 1989) should have been adopted for assessing exercise enjoyment during exercise. However, the PACES was employed to facilitate comparisons of enjoyment “during” and “after” the sessions. Nonetheless, no differences in enjoyment “during” and “after” the exercise session were observed.

Furthermore, the questionnaire was completed between intervals 2 and 3 of the high intensity interval exercise session, where the intensity and the effort are low. Hence, completing the questionnaire during one or each high intensity interval, when the effort is higher, may have generated different enjoyment responses. However, the 18-item scale was considered too comprehensive to be employed during intervals of high effort. Therefore, filling out the questionnaire following 55% completion of the exercise session seemed appropriate and feasible.

Previous studies of similar designs employed the Feeling Scale (Stanley and Cumming, 2010), a one-item scale intended to measure affective valence (positive to negative) (Hardy and Rejeski, 1989). Interestingly, a decline in positive affective valence responses to increased exercise intensity has been observed (Parfitt et al., 2006; Rose and Parfitt, 2007; Sheppard and Parfitt, 2008; Oliveira et al., 2018). A similar pattern may have been observed in this study if affective valence was measured. However, the aim of this study was to explore the potential for high exercise enjoyment following interval durations of 4 min, which was confirmed by our data. Future studies may reveal potential differences in affective valence for high intensity intervals over 3 min compared with other exercise modalities.

### Strengths

To our knowledge, this study is the first to assess the exercise enjoyment of high intensity interval exercise with interval set durations of 4 min. Considering the greater improvements in cardiorespiratory fitness associated with 4 min intervals (Helgerud et al., 2007; Bacon et al., 2013) and the findings of similar enjoyment in 4 min high intensity intervals, longer interval set durations cannot be disregarded when precribing high intensity interval exercise as preventive medicine. Moreover, the exercise conditions in this study were iso-caloric in order to avoid any confouding effects of energy expenditure on exercise enjoyment. At the same time, the duration of the exercise sessions differed, and because lack of time is reported to be one of the reasons underlying low exercise adherence (Leslie et al., 1999; Stutts, 2002), the potential implications of exercise duration, and accumulated percieved exertion from intensity and duration as discussed above, on the results of the present study cannot be ruled out.

### Limitations

This study included eight participants, and one participant withdrew, resulting in seven participants for the final analyses. In general, seven participants can be regarded as a low number of individuals for causality inferences, and the small sample size may have masked different enjoyment effects between the two exercise modalities. Thus, a statistical type 2 error cannot be conclusively ruled out. At the same time, a previous study in the field successfully included few (*n* = 8) participants using a similar crossover design (Bartlett et al., 2011), and the calculated effect sizes for exercise enjoyment in the two conditions of the present study are considered medium, indicating some noticeable effects estimations (Cohen, 1992). Moreover, in order to calculate statistical power prior to an experiment, a clinically relevant difference must be assumed. To our knowledge, no clinically relevant difference exists for the PACES questionnaire. A potential future research direction may be to clarify a potential clinically relevant difference in PACES score for exercise adherence, preferably from studies investigating the long-term exercise adherence effects of different exercise modalities. Nevertheless, the results presented here seem plausible, both in terms of the results on exertion as well as the exercise enjoyment findings from a psychological-physiological interaction approach, as anticipated based on the known anaerobic contribution to affective responses (Hall et al., 2002; Oliveira et al., 2018). However, considering the unknown clinical relevance of the PACES questionnaire for exercise adherence, this study can be regarded as a pilot study to inform future research.

To ensure iso-caloric exercise conditions, we used percentage of HR_*max*_ as our intensity prescription (Helgerud et al., 2007). However, it is previously demonstrated that similar relative percentage of HR_*max*_ may not reflect similar blood lactate values between individuals (Meyer et al., 1999). Thus, the included participants in our study may have accumulated different blood lactate values when performing the same exercise session. As our study aimed to elucidate potential enjoyment differences from exercise sessions with different anaerobic contributions, prescribing the intensity as percentage of individual anaerobic treshold may have been more appropriate. Thus, we may have missed potential differences in enjoyment due to different interindividual anaerobic contributions. Future research that aims to assess enjoyment in relation to anaerobic metabolism may benefit from prescribing the exercise intensity as percentage of individual anaerobic threshold.

The participants included in the present study displayed a median peak oxygen uptake of 53.2 ml kg^–1^ min^–1^, which is comparable to Norwegian young adults (Aspenes et al., 2011) but slightly higher than that of American young adults (Kaminsky et al., 2015). However, the latest meta-analysis reported no linear association between cardiorespiratory fitness and enjoyment of aerobic exercise (Oliveira et al., 2018), indicating that exercise enjoyment may not be dependent on an individual’s initial fitness level. Nevertheless, as the aim of this study was to assess the exercise enjoyment of 4 min intervals, and because long interval set durations (≥3 min) provide greater cardiorespiratory fitness improvements compared with high intensity intervals of shorter durations or lower intensity exercise (Bacon et al., 2013), the findings from the present study could be considered applicable across populations of different cardiorespiratory fitness levels.

Finally, although not measured in the present study, the participants included in this study defined themselves as recreationally active individuals. As the majority of Norwegian adults do not fulfill current global recommendations for physical activity (Hansen et al., 2012, 2019), the present study may not be applicable to the large inactive population, which may be considered the population that could derive the greatest benefit from engaging in exercise (Blair et al., 1995; Leslie et al., 1999; Myers et al., 2002; Stutts, 2002; Lear et al., 2017). Future studies assessing exercise enjoyment across different physical activity levels are warranted.

## Conclusion

Similar exercise enjoyment was reported following four high intensity intervals of 4 min compared with a moderate intensity CE session in this randomized crossover study with iso-caloric conditions. Thus, if enjoyment is a mediating factor for engaging in exercise, one should expect a similar exercise adherence probability following high intensity 4 min intervals and CE of moderate intensity when prescribing aerobic exercise as preventive medicine.

## Data Availability

All datasets generated for this study are included in the manuscript and/or the Supplementary Data Sheets 1, 2.

## Author Contributions

ES conceived and designed the study, performed the statistical analyses, and in charge of the writing process. SP conceived and designed the study, and wrote the manuscript. TomH, TorH, KR, and SAP wrote and reviewed the manuscript.

## Conflict of Interest Statement

The authors declare that the research was conducted in the absence of any commercial or financial relationships that could be construed as a potential conflict of interest.
