# Supplementary material for: High Intensity Long Interval Sets Provides Similar Enjoyment as Continuous Moderate Intensity Exercise. The Tromsø Exercise Enjoyment Study
Source: Front Psychol. 2019 Aug 6;10:1788. doi: 10.3389/fpsyg.2019.01788 (PMC6691159; doi:10.3389/fpsyg.2019.01788)
Supplement: Supplementary file 1 [file Table_1.docx]

Supplementary Material

# Supplementary Data

Supplementary file 1: Zip-file containing the SPSS file with the data supporting the conclusion of this study.

Supplementary file 2: Zip-file containing the “Read me”-file to explain the variables in the SPSS-file.

# Supplementary Tables

**Supplementary Table 1**:

**Table 1:** The translated version of the PACES questionnaire:

«Vennligst ranger hvordan du føler akkurat nå om den fysiske aktiviteten du har gjort/gjør:»

|  | 1 | 2 | 3 | 4 | 5 | 6 | 7 |  |
| --- | --- | --- | --- | --- | --- | --- | --- | --- |
| Jeg nyter det |  |  |  |  |  |  |  | Jeg hater det |
| Jeg føler kjedsomhet |  |  |  |  |  |  |  | Jeg føler meg interessert |
| Jeg misliker det |  |  |  |  |  |  |  | Jeg liker det |
| Jeg finner det behagelig |  |  |  |  |  |  |  | Jeg finner det ubehagelig |
| Jeg finner det altoppslukende |  |  |  |  |  |  |  | Jeg finner det ikke altoppslukende |
| Det er ikke morsomt i det hele tatt |  |  |  |  |  |  |  | Det er virkelig morsomt |
| Jeg finner det energigivende |  |  |  |  |  |  |  | Jeg finner det slitsomt |
| Det gjør med deprimert |  |  |  |  |  |  |  | Det gjør meg glad |
| Det er veldig hyggelig |  |  |  |  |  |  |  | det er veldig uhyggelig |
| Jeg føler meg fysisk bra mens jeg gjør det |  |  |  |  |  |  |  | Jeg føler meg fysisk dårlig mens jeg gjør det |
| Der er veldig styrkende |  |  |  |  |  |  |  | Det er ikke styrkende i det hele tatt |
| jeg er veldig frustrert av det |  |  |  |  |  |  |  | Jeg er ikke frustrert av det i det hele tatt |
| Det er veldig gledelig |  |  |  |  |  |  |  | Det er ikke gledelig i det hele tatt |
| Det er veldig spennende |  |  |  |  |  |  |  | Det er ikke spennende i det hele tatt |
| Det er ikke stimulerende i det hele tatt |  |  |  |  |  |  |  | Det er veldig stimulerende |
| Det gir meg en sterk følelse av gjennomføringskraft |  |  |  |  |  |  |  | det gir meg ingen følelse av gjennomføringskraft |
| Det er veldig forfriskende |  |  |  |  |  |  |  | Det er ikke forfriskende i det hele tatt |
| Jeg ville heller gjøre noe annet |  |  |  |  |  |  |  | Det var ikke noe annet jeg heller vil gjøre |

**Supplementary Table 2:**

|  |  |
| --- | --- |
| Age (yr) | 23.4 ± 2.0 |
| Weight (kg) | 72.5 ± 4.9 |
| Height (cm) | 174.4 ± 6.4 |
| BMI (kg·m^-2^) | 23.9 ± 1.0 |
| Peak Oxygen Uptake (ml·kg^-1^·min^-1^) | 52.1 ± 8.7 |

**Table 2.** Participants´ characteristics (n=7). Data is mean ± standard deviation

|  | **High Intensity Interval Exercise** | **Moderate Intensity Continuous Exercise** |
| --- | --- | --- |
| Rating of Perceived Exertion  Pooled (n=14) | 16.2±1.5* | 9.9±1.2 |
| During exercise (n=7) | 15.4±1.3*# | 9.8±1.2 |
| After exercise (n=7) | 17.0±1.3* | 10.0±1.3 |
| Physical Activity Enjoyment Scale  Pooled (n=14) | 95.5±12.6 | 92.8±15.3 |
| During exercise (n=7) | 94.4±12.9 | 91.1±16.3 |
| After exercise (n=7) | 96.6±13.2 | 94.4±15.4 |

**Supplementary Table 3:**

**Table 3.** Exercise enjoyment and rating of perceived exertion during and after high intensity interval exercise and continuous exercise.

Data are shown as mean ± standard deviation. *Significant difference between high intensity interval exercise and continuous exercise <0.05. #Significant difference between during and after exercise in the same exercise mode <0.05

**
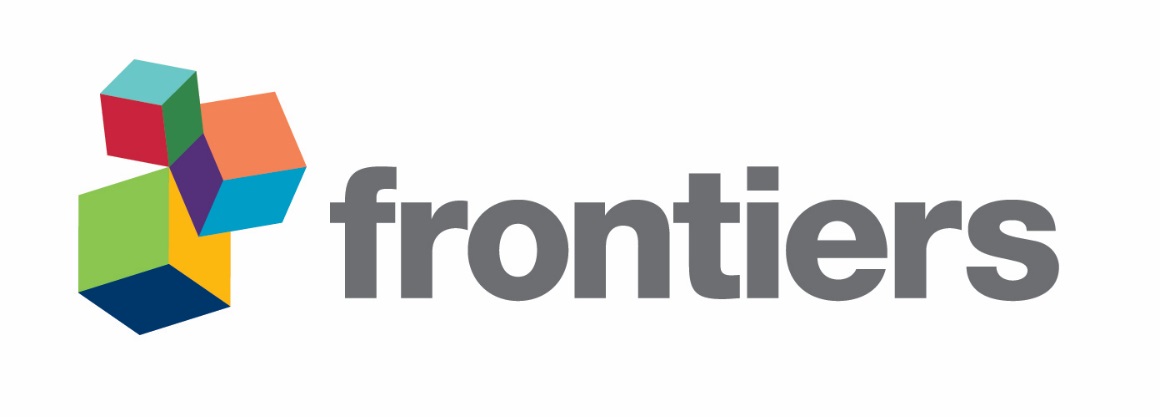
**
